# Identification of common microRNA between COPD and non-small cell lung cancer through pathway enrichment analysis

**DOI:** 10.1186/s12863-021-00986-z

**Published:** 2021-10-12

**Authors:** Amirhossein Fathinavid, Mohadeseh Zarei Ghobadi, Ali Najafi, Ali Masoudi-Nejad

**Affiliations:** 1 Laboratory of Systems Biology and Bioinformatics (LBB), Department of Bioinformatics, Kish International Campus, University of Tehran, Kish Island, Iran; 2 Laboratory of Systems Biology and Bioinformatics (LBB), Institute of Biochemistry and Biophysics, University of Tehran, Tehran, Iran; 3 Molecular Biology Research Center, System Biology and Poisoning Institute, Tehran, Iran

**Keywords:** COPD, Non-small cell lung Cancer, miRNA, Pathway analysis

## Abstract

**Background:**

Different factors have been introduced which influence the pathogenesis of chronic obstructive pulmonary disease (COPD) and non-small cell lung cancer (NSCLC). COPD as an independent factor is involved in the development of lung cancer. Moreover, there are certain resemblances between NSCLC and COPD, such as growth factors, activation of intracellular pathways, as well as epigenetic factors. One of the best approaches to understand the possible shared pathogenesis routes between COPD and NSCLC is to study the biological pathways that are activated. MicroRNAs (miRNAs) are critical biomolecules that implicate the regulation of several biological and cellular processes. As such, the main goal of this study was to use a systems biology approach to discover common dysregulated miRNAs between COPD and NSCLC, one that targets most genes within common enriched pathways.

**Results:**

To reconstruct the miRNA-pathways for each disease, we used the microarray miRNA expression data. Then, we employed “miRNA set enrichment analysis” (MiRSEA) to identify the most significant joint miRNAs between COPD and NSCLC based on the enrichment scores. Overall, our study revealed the involvement of the targets of miRNAs (such as has-miR-15b, hsa-miR-106a, has-miR-17, has-miR-103, and has-miR-107) in the most important common biological pathways.

**Conclusions:**

According to the promising results of the pathway analysis, the identified miRNAs can be utilized as the new potential signatures for therapy through understanding the molecular mechanisms of both diseases.

**Supplementary Information:**

The online version contains supplementary material available at 10.1186/s12863-021-00986-z.

## Background

Chronic obstructive pulmonary disease (COPD) is a lung-related disease specified by the continuous respiratory symptoms and boosted inflammatory response owing to harmful gases and particles [1, 2]. On the one hand, COPD raises oxidative stress leading to DNA damage, chronic exposure, repression of the DNA repair mechanisms, and cellular proliferation [3]; on the other hand, lung cancer as the fifth cancer leading to global mortality is usually classified into two main histologic types: non-small cell lung cancer (NSCLC) and small cell lung cancer (SCLC) [4]. Moreover, the mutations in oncogenes can also lead to lung cancer, and as a result, cell proliferation and forming a tumor [4, 5]. Furthermore, cell proliferation and unsuppressed cell growth are the known characteristics of cancer progression in which several genes and proteins are involved, especially, the kinases and kinase receptors [6].

The rate of lung cancer in patients with COPD is nearly five times more than that of smokers without COPD [7]; besides, the overlap between COPD and lung cancer can be due to joint genetic susceptibility as well as the smoking-related processes [8]. COPD is recognized as an autonomous risk factor for lung cancer, particularly for NSCLC as the most prevalent lung cancer type [9]. Both COPD and NSCLC are mostly caused by cigarette smoking [8] through inducing inflammation and oxidative stress in the lung [10]. Some common processes would contribute to the development of COPD and lung cancer in patients, such as abnormal immunity, cell proliferation, apoptosis, and chromatin modifications [11].

MicroRNAs are a category of functional non-coding RNAs containing 20 ~ 24 nucleotides, what negatively governs mRNA stability and/or suppresses mRNA translation through binding to the 3′ untranslated region [12, 13]. The role of miRNAs in a wide range of cellular process, including proliferation, cell cycle, differentiation, apoptosis, and metastasis has been reported [13]. MiRNAs are involved in the initiation and development of disparate cancer types, while they are dysregulated in many cancers. Moreover, the alteration in the mRNA expression levels is also correlated with several diseases such as cardiovascular diseases, immunity- or inflammation-related diseases, and COPD [14, 15]. Furthermore, miRNAs function as oncogenes or tumor suppressors through regulating their target genes. It should be noted that miRNAs have great potential to be used as therapeutic targets, therefore, the determination and visualization of their positions in the regulatory pathways will be helpful in the development of novel medications [16]. If a miRNA is related to the physiological process, it certainly regulates a gene or multiple genes in a corresponding pathway.

There are many common pathways that are activated in COPD and NSCLC [17]. Athyros and colleagues, for one, found the impairment of several steps in the reverse cholesterol transport pathway via systematic inflammation in COPD [18]. Moreover, Aldehydes identified the elevation of histone 3 phosphorylation in cigarette smokers via the activation of proliferative pathways, including the phosphatidylinositol-3 kinase (PI3K) / protein kinase B (PKB/Akt) [19] and MAPK pathway [20, 21]. The KEGG database is a series of biological pathways wherein many genes, proteins and other products are involved; however, the information about miRNAs is not mentioned in them. Cong Pian et al. [21] designed a new pathway database with the aid of KEGG plus miRNAs and integrated the human miRNA-target interactions with KEGG pathways using the hypergeometric test. Furthermore, C. Brinkrolf et al. [22] introduced a platform called VANESA for reconstructing, visualizing, and analyzing biological networks, to predict human miRNAs that may be co-expressed with genes involved in the KEGG pathway.

The aim of this study is to identify the most significant miRNAs as the new biomarkers which are common between COPD and NSCLC via analyzing the shared pathways between both diseases. To this aim, we considered two miRNA datasets related to COPD and NSCLC and normalized each dataset; then, we enriched both datasets to detect those pathways that contained more target genes for each miRNA list.

**Fig. 1:**
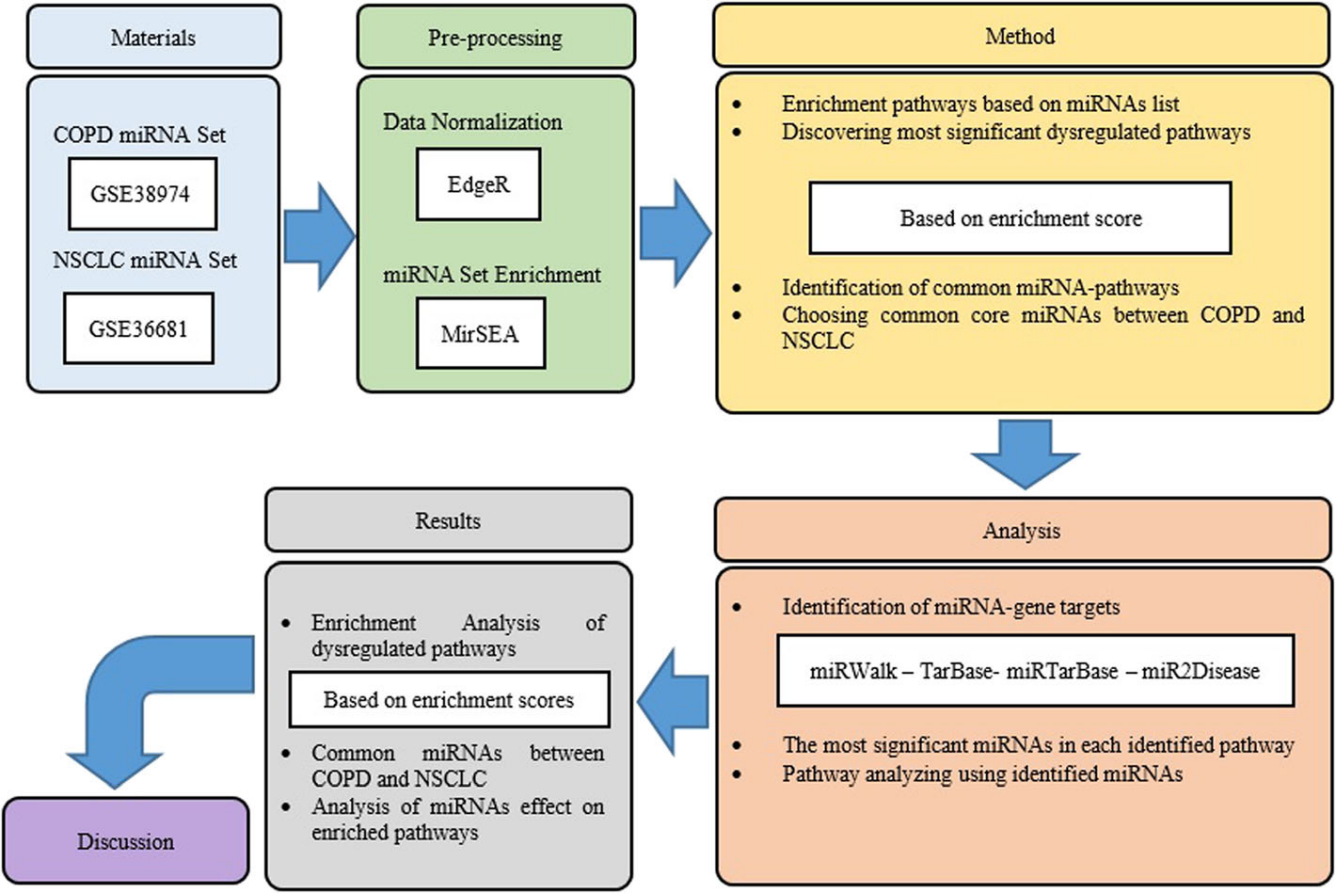
The workflow of steps performed in this study. This scheme shows that after collecting miRNA expression profiles, pre-processing was individually performed for each dataset, and then, the enrichment miRNA-pathways were utilized to discover dysregulated pathways though miRNA sets. Those common miRNAs that had the most effects on the enriched pathways on the basis of enrichment scores were selected, and the target genes were extracted from target prediction databases for common miRNAs between COPD and NSCLC. At the end, the pathways analysis was performed

Thus, we detected those miRNAs that targeted more genes within the shared pathways and had more metabolic and genetic impact on the enriched pathways; then, we introduced the common pathways with the common miRNAs between COPD and NSCLC; and finally, we analyzed the enriched miRNA-pathway sets by identifying the number of target genes for each miRNAs that contributed in a specific pathway. To have an overall view, the workflow of the different steps is visualized in Fig. 1.

**Table 1:** Top 10 down-regulated pathways in COPD

Pathway	SIZE	ES	NES	Mir \%	Signal
KEGG_OOCYTE MEIOSIS	54	− 0.76904	−2.6434	0.0726	0.502
KEGG_REGULATION OF ACTIN CYTOSKELETON	85	− 0.71525	−2.5143	0.0826	0.45
KEGG_CELL CYCLE	124	−0.66193	−2.4711	0.125	0.46
KEGG_RENAL CELL CARCINOMA	108	−0.67663	−2.4694	0.115	0.447
KEGG_NON-SMALL CELL LUNG CANCER	105	−0.64361	−2.407	0.121	0.464
KEGG_ERBB_SIGNALING_PATHWAY	97	−0.58372	−2.3976	0.115	0.414
KEGG_P53 SIGNALING PATHWAY	92	−0.66285	−2.396	0.115	0.455
KEGG_VEGF_SIGNALING_PATHWAY	68	−0.59528	−2.3986	0.108	0.415
KEGG_TGF_BETA_SIGNALING_PATHWAY	66	−0.65517	−2.3876	0.113	0.453
KEGG_WNT SIGNALING PATHWAY	32	−0.73266	−2.383	0.0657	0.539

## Results

This study presents common miRNA biomarkers between COPD and NSCLC of pre-processed datasets via miRNA-pathway set enrichment analysis and highlights those pathways with more target genes of miRNAs associated with COPD and NSCLC. As such, it specifies the most significant miRNAs or core miRNAs using analyzed pathways. In addition, it assesses the most significant pathways by affecting the core miRNAs on their targets as the components in the pathways. In the meanwhile and as a final step, this study has performed a literature-based search to study the identified miRNA biomarkers on the common pathways.

### MiRNA datasets

To construct the expression matrices for all samples, we removed zero values from both datasets. Eventually, the total number of miRNAs after normalization was equal to 1308 and 1145 miRNAs for COPD and NSCLC, respectively; which were considered for further analysis.

The workflow of steps performed in this study. This scheme shows that after collecting miRNA expression profiles, pre-processing was individually performed for each dataset, and then, the enrichment miRNA-pathways were utilized to discover dysregulated pathways though miRNA sets. Those common miRNAs that had the most effects on the enriched pathways on the basis of enrichment scores were selected, and the target genes were extracted from target prediction databases for common miRNAs between COPD and NSCLC. At the end, the pathways analysis was performed.

### Enrichment analysis and identification of dysregulated pathways

The results of miRNA set enrichment analysis revealed the pathways regulated by each miRNA in each disease. We identified 149 significant enriched pathways in COPD (1 up-regulated and 148 down-regulated pathways) and 146 significant enriched pathways in NSCLC (72 up-regulated and 74 down-regulated pathways). Among all enriched pathways, similar pathways were found between down-regulated pathways in COPD and up-regulated pathways in NSCLC. In Tables 1 and 2, we only demonstrated the top 10 significant enriched pathways for COPD and NSCLC, respectively. In these tables, size of pathways based on the number of contributed features (SIZE), pathways’ enrichment scores before and after running enrichment peak (ES and NES), percentage of miRNA list before running enrichment peak (Mir%), and the enrichment signal strength are represented in the columns. The full list of common pathways between both diseases is shown in Table S2 and S3 for COPD and NSCLC, respectively.

By comparing the enrichment results, we selected 7 common dysregulated pathways with different regulations in COPD and NSCLC, including non-small cell lung cancer, cell cycle, P53 signaling pathway, VEGF signaling pathway, TGF beta signaling pathway, WNT signaling pathway, and ERBB signaling pathway.

### Common miRNAs between COPD and NSCLC

Table S1 shows the enriched pathways and common core miRNAs between COPD and NSCLC in each pathway in such a way that these miRNAs were at least common between the two pathways. For better recognition, in Table S1, each miRNA is highlighted with a color scale from Green to Yellow to show well the degree of the replication of the miRNAs in all enriched pathways.

Moreover, to detect significant miRNAs among all pathways, the average enrichment scores of each miRNA for all enriched pathways as well as the mean score of core miRNAs within each pathway were calculated and shown in Table 4. The zero value in each cell means that the miRNA was not found in that pathway.

**Table 2:** Top 10 up-regulated pathways in NSCLC

Pathway	SIZE	ES	NES	Mir \%	Signal
KEGG_PRIMARY IMMUNODEFICIENCY	10	0.83905	1.8172	0.00556	0.303
KEGG_P53 SIGNALING PATHWAY	100	0.49347	1.6518	0.127	0.325
KEGG_ERBB SIGNALING PATHWAY	90	0.47255	1.6308	0.102	0.285
KEGG_NON-SMALL CELL LUNG CANCER	86	0.48247	1.6235	0.089	0.253
KEGG_CELL CYCLE	116	0.4208	1.5168	0.106	0.267
KEGG_APOPTOSIS	67	0.46177	1.4878	0.132	0.343
KEGG_WNT SIGNALING PATHWAY	87	0.40255	1.382	0.124	0.321
KEGG_PRION DISEASES	27	0.51335	1.3818	0.113	0.273
KEGG_VEGF SIGNALING PATHWAY	62	0.37869	1.3925	0.128	0.277
KEGG_TGF BETA SIGNALING PATHWAY	60	0.37867	1.2741	0.0209	0.16

**Fig. 2:**
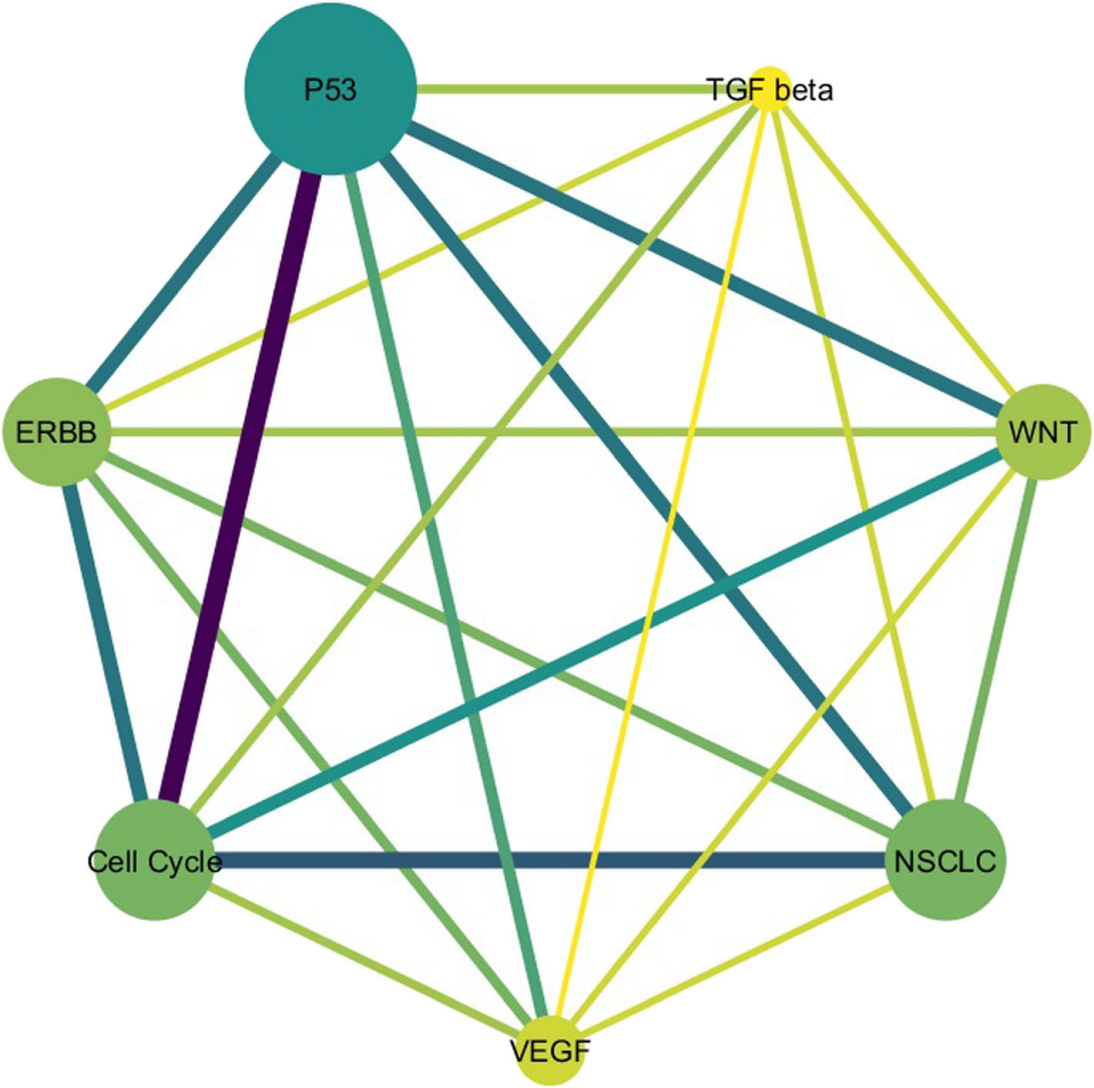
The network of common pathways. Each node represents the pathway, the size and the color depth of each node indicate the number of common core miRNAs between COPD and NSCLC in that pathway; also, the thickness of an edge in this network represents the number of shared miRNAs between the two pathways. P53 signaling, cell cycle, and non-small cell lung cancer pathways have the highest number of common miRNAs between COPD and NSCLC, in which the number of core miRNAs in p53 signaling, cell cycle, and non-small cell lung cancer pathways are 15, 15, and 10, respectively

A network of common pathways is also shown in Fig. 2, in which each node in this network represents the pathway and each edge between two nodes indicates that there are common miRNAs between two pathways.

**Table:** Table 3

KEGG_CELL_CYCLE	KEGG_ERBB_SIGNALING	KEGG_P53_SIGNALING	KEGG_TGF_BETA_SIGNALING	KEGG_VEGF_SIGNALING	KEGG_WNT_SIGNALING	KEGG_NON_SMALL_CELL_LUNG_CANCER
hsa-miR-107	hsa-let-7b	hsa-miR-107	hsa-miR-133a	hsa-miR-17	hsa-miR-17	hsa-miR-103
hsa-miR-654-3p	hsa-let-7c	hsa-let-7d	hsa-let-7c	hsa-miR-107	hsa-miR-15a	hsa-let-7c
hsa-let-7d	hsa-miR-1	hsa-miR-654-3p	hsa-miR-1	hsa-let-7b	hsa-let-7c	hsa-miR-17
hsa-miR-1	hsa-miR-654-3p	hsa-miR-1	hsa-miR-455-3p	hsa-let-7d	hsa-miR-133a	hsa-miR-455-3p
hsa-miR-455-3p	hsa-miR-193a-3p	hsa-miR-361-5p	hsa-let-7d	hsa-miR-16	hsa-miR-103	hsa-miR-106a
hsa-miR-17	hsa-miR-107	hsa-let-7c		hsa-miR-193a-3p	hsa-let-7b	hsa-miR-107
hsa-miR-15b	hsa-miR-17	hsa-miR-15a		hsa-miR-133a	hsa-miR-1285	hsa-miR-1285
hsa-miR-103	hsa-let-7d	hsa-miR-133a			hsa-miR-1	hsa-miR-133a
hsa-miR-1285	hsa-miR-133b	hsa-miR-15b			hsa-let-7e	hsa-miR-133b
hsa-let-7b	hsa-miR-106a	hsa-let-7b				hsa-miR-15b
hsa-let-7e		hsa-miR-103				hsa-miR-203
hsa-miR-106a		hsa-miR-106a				
hsa-miR-361-5p		hsa-miR-17				
hsa-miR-203		hsa-miR-16				
hsa-let-7c		hsa-miR-1285

Next, since we aimed to clarify the significant miRNAs in common pathways between COPD and NSCLC, we selected the most significant enriched pathways based on two factors: the calculated scores (Table 4) and the number of core miRNAs (Fig. 2). Given pathways, including cell cycle, P53 signaling, non-small cell lung cancer, VEGF signaling, ERBB signaling, WNT signaling, and TGF beta signaling pathways in KEGG had the mean enrichment scores: − 0.0628, − 0.0554, − 0.04016, − 0.0306, − 0.0208, − 0.0201, and − 0.0079, respectively. The results showed that the average number of NES in all pathways for COPD that have more pathways than NSCLC were almost equal to − 2, this means that the NES lower than − 2 could be meaningful in biology for COPD. But for NSCLC, the changes of NES were almost stable (0 ∙ 8 ≤ *NES* ≤ 1 ∙ 1); thus, we considered those pathways with the average of NES less than − 2 for COPD and found the common significant pathways between both diseases. Moreover, the number of core miRNAs in each pathway, as the second factor, was determined to be equal to 15, 15, 11, 10, 9, 7, and 5 for p53 signaling, cell cycle, non-small cell lung cancer, ERBB signaling, WNT signaling, VEGF signaling, and TGF beta signaling pathways, respectively. Finally, the pathways comprising the highest average enrichment scores along with high number of common core miRNAs were selected. Therefore, three pathways including cell cycle, non-small cell lung cancer, and p53 signaling pathways were detected as the most significant pathways. As a further note, the number of shared miRNAs between p53 signaling and cell cycle pathways was 12, the common miRNA numbers between cell cycle and NSCLC pathways was 9, and the number of shared miRNAs between p53 signaling and NSCLC pathways was 8.

**Table 4:** List of core miRNAs that are common among all pathways. Mean Score column is the average of all miRNA enrichment scores among all pathways, and Mean Score raw is the average of enrichment scores about all miRNAs in a specific pathway

miRNAs	CELL CYCLE	ERBB SIGNALING	P53 SIGNALING	TGF_BETA SIGNALING	VEGF SIGNALING	WNT SIGNALING	NON_SMALL CELL LUNG CANCER	Mean Score
hsa-miR-106a	0.0495	0.1025	0.067	0	0	0	0.1145	0.083375
hsa-miR-15b	0.076	0	0.085	0	0	0	0.121	0.094
hsa-miR-17	0.0775	0.1265	0.0885	0	0.079	−0.02	0.074	0.070917
hsa-miR-103	0.015	0	0.0245	0	0	0.051	0.0612	0.037925
hsa-miR-107	0.0075	0.033	0.007	0	0.0115	0	0.052	0.0222
hsa-miR-133b	0	0.022	0	0	0	0	0.002	0.012
hsa-let-7d	0.059	0.03	0.0515	−0.08	0.03	0	0	0.0181
hsa-miR-193a-3p	0	0.019	0	0	0.02	0	0	0.0195
hsa-miR-16	0	0	−0.0625	0	−0.0775	0	0	−0.07
hsa-miR-455-3p	0.0455	0	0	0.082	0	0	0.052	0.059833
hsa-miR-15a	0	0	−0.032	0	0	−0.006	0	−0.019
hsa-miR-361-5p	−0.0715	0	−0.0225	0	0	0	0	−0.047
hsa-let-7e	−0.0915	0	0	0	0	−0.065	0	−0.07825
hsa-let-7c	−0.1535	−0.0845	− 0.1355	−0.014	0	−0.001	− 0.0645	−0.0755
hsa-miR-133a	0	0	−0.1325	−0.01185	− 0.1448	−0.075	− 0.075	−0.08783
hsa-let-7b	−0.1315	−0.1605	− 0.1195	0	− 0.1325	−0.028	0	−0.1144
hsa-miR-1285	−0.2455	0	−0.247	0	0	0.1155	−0.218	−0.14875
hsa-miR-1	−0.2115	−0.135	− 0.226	−0.01585	0	−0.1525	0	−0.14817
hsa-miR-654-3p	−0.18	−0.1825	− 0.178	0	0	0	0	−0.18017
hsa-miR-203	−0.188	0	0	0	0	0	−0.561	−0.3745
Mean Score	−0.0628	−0.0208	− 0.0554	−0.0079	− 0.0306	−0.0201	− 0.04016	

### Significant miRNAs in the selected enriched pathways

**Fig. 3:**
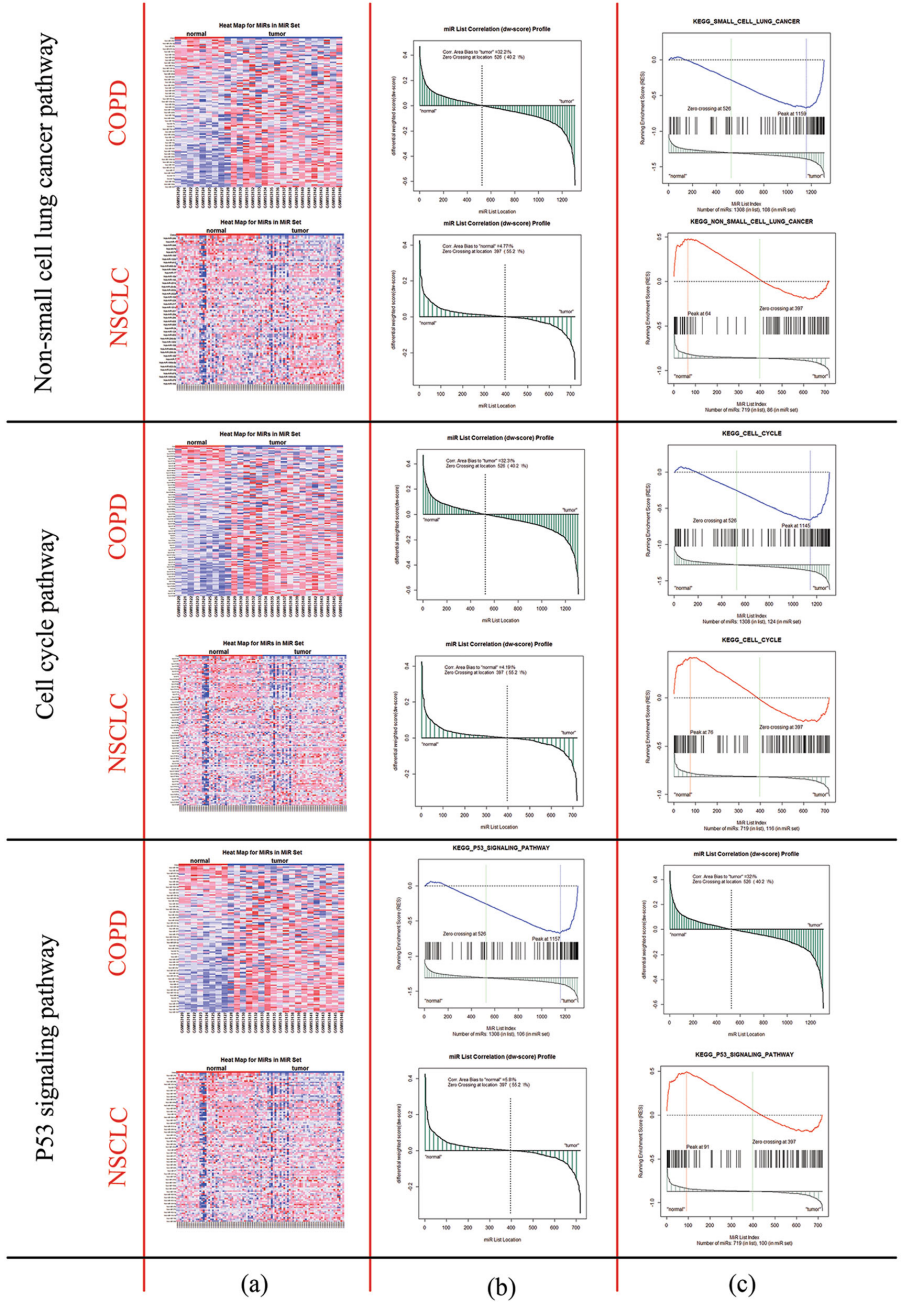
Results of miRNA set enrichment analysis in COPD and NSCLC for non-small cell lung cancer, cell cycle, and p53 signaling pathways. MiRSEA performs differential expression analysis for miRNAs based on differential weighted scores (**a**); integrates the differential expression level of miRNAs and miRNA-pathway weights, calculates miRScore, and creates a ranked list of miRNAs. Then, it maps miRNAs in the pathway to the ranked list and calculates the miRNA enrichment score for each pathway and miRNA correlation profiles (**b**); after calculating the enrichment score, MiRSEA prioritizes a pathway by FDR and the running miRNAs enrichment score for the pathway results (**c**)

In Fig. 3, the results for the differential expression level of miRNA set (miRNA-pathway) along with weighted miRNA correlation and ranked miRNA list based on miRScores in non-small cell lung cancer, cell cycle, and p53 signaling pathways are visualized.

**Fig. 4:**
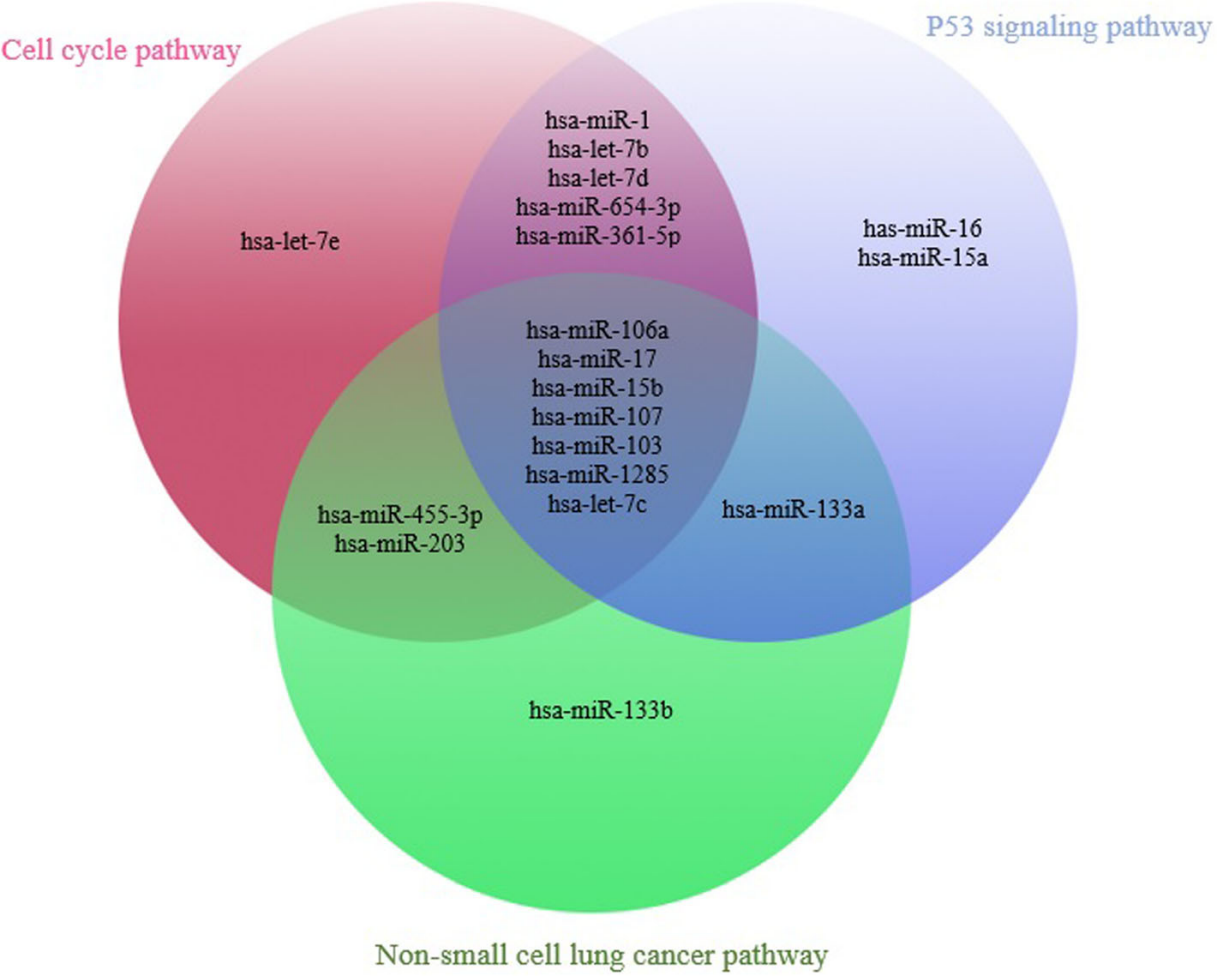
Significant core miRNAs among three enriched pathways based on average enrichment scores

Through the selected enriched pathways, we determined core miRNAs which were shared among these pathways and also had higher average of enrichment scores than the other miRNAs. In Fig. 4, the most significant common core miRNAs among three selected pathways are depicted; furthermore, the overlapping core miRNAs of cell cycle, p53 signaling, and non-small cell lung cancer pathways are shown as well.

**Fig. 5:**
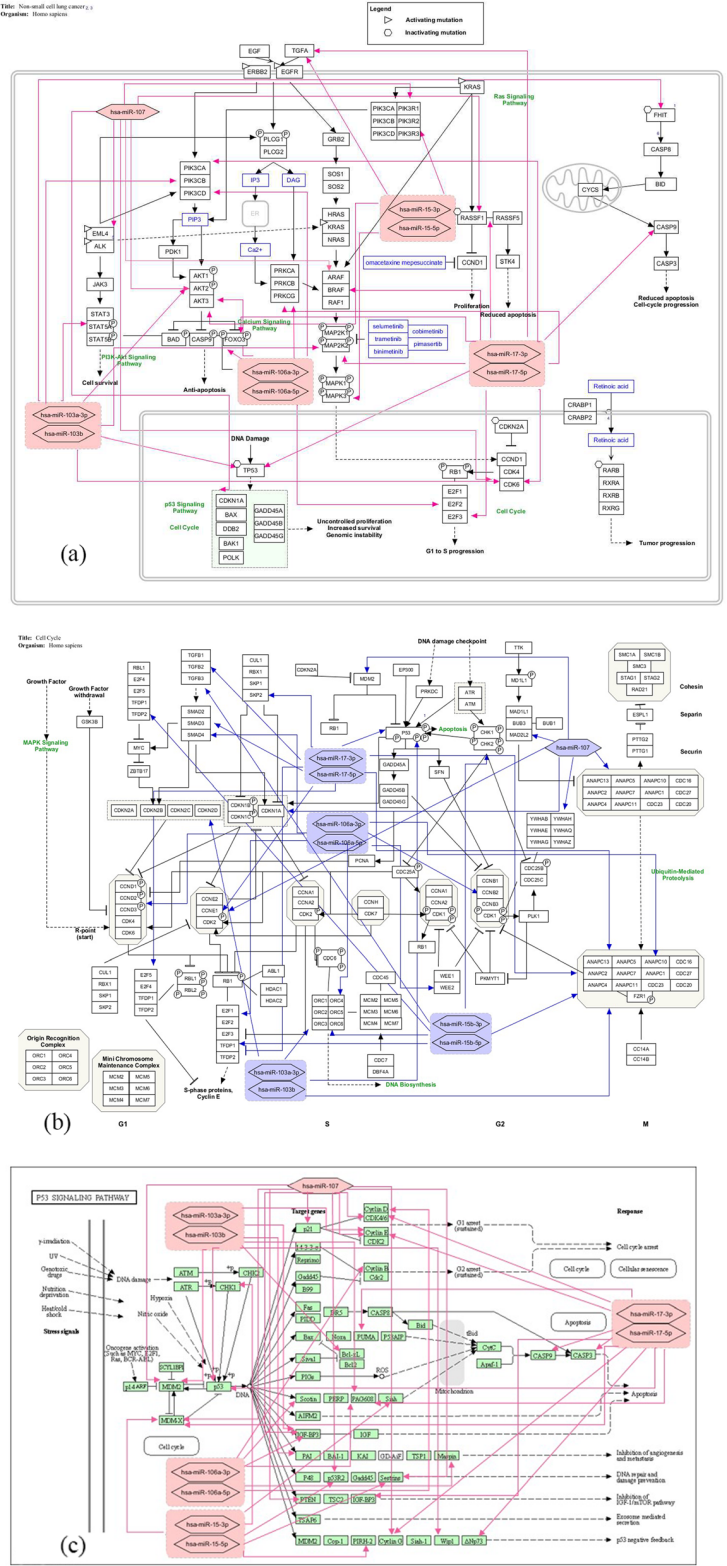
Converted non-small cell lung cancer (**a**) cell cycle (**b**) and p53 signaling (**c**) pathways in KEGG. The target genes of has-miR-15b, hsa-miR-106a, has-miR-17, has-miR-103 and has-miR-107 as the most significant core miRNAs are identified and then these miRNAs are mapped to the pathways

As a result, the has-miR-15b, hsa-miR-106a, has-miR-17, has-miR-103 and has-miR-107 were selected as the most significant miRNAs with 0.094, 0.083, 0.07, 0.037, and 0.022, respectively, to be the mean of their enrichment scores among all pathways. Afterward, the target genes of the most significant miRNAs in each pathway were identified and the targets mapped to selected pathways. The converted pathways are demonstrated in Fig. 5a, b and c.

## Discussion

Until now, diverse functions have been introduced for miRNAs including inflammation, development of airway epithelial cells, stress responses, and formation of pulmonary surfactant; also, miRNAs have critical roles in the disease’s progression [23]. Moreover, COPD as a progressive and incompletely reversible disease leads to chronic inflammation due to considerable dysregulation of the immune system. Some studies have been conducted to reveal the common pathogenesis mechanisms between COPD and NSCLC [24–27]. Herein, we have investigated common actuated pathways in both diseases and the role of miRNAs in them. Although we have investigated the most significant common biological pathways between both diseases, our main goal has been to identify the common miRNAs through a pathway enrichment analysis method, what has not been employed before.

To this aim, we first identified DEMs within each pathway as miRNA-pathway; and next, we considered the miRNAs which had more target genes in that pathway. In addition, to avoid the false-positive miRNA-target interactions, we considered the experimentally validated miRNA-target interaction using miRNA-target prediction databases. To select the most significant enriched pathways, a threshold was considered based on the number of DEMs that enriched the pathways. After pathway enrichment, we analyzed three pathways, including non-small cell lung cancer, cell cycle, and p53 signaling pathways as the remarkable enriched pathways based on average ES and their common involved miRNAs, including has-miR-15b, hsa-miR-106a, has-miR-17, has-miR-103 and has-miR-107. These pathways were down-regulated in COPD and up-regulated in NSCLC. The aforementioned miRNAs have been demonstrated to be associated with COPD and lung cancer [28–37]. The point is that though miRNAs’ roles are known in COPD and NSCLC, understanding their functions in common significant pathways may shed light on the pathogenic mechanism of both diseases and develop new therapeutic targets.

MiR-107 is a tumor suppressor that targets the epidermal growth factor receptor (EGFR). The deregulation of EGFR has been observed in multiple types of cancers, including NSCLC, while frequent EGFR protein overexpression was observed in NSCLC and COPD [38, 39]. Not only EGFR facilitates proliferative signaling through downstream signaling pathways, i.e. PI3K/AKT/mTOR and RAS/ERK, EGFR signaling pathway is one of the activated pathways in lung cancer and employs downstream RAS or ERK pathways to direct proliferative signaling for lung cancer cells [40]. Moreover, the down-regulation of miR-107 may cause cell cycle and proliferation due to the up-regulation of CDK6 (also targeted by miR-103) and CDK8 and metastasis and tumor growth because of the up-regulation of BDNF as well as indirect regulating of the P13K/AKT signaling pathway [40, 41]. The specific binding of BDNF to tropomyosin-related receptor kinase B (TrkB) results in the activation of several downstream pathways such as RAS/ERK, PLC/PKC, PI3K/AKT, JAK/STAT, and AMPK/ACC [40]. However, miR-107 was up-regulated in COPD, which might cause down-regulation of its targets leading to down-regulation of downstream pathways of cell cycle and non-small cell lung cancer pathways. Furthermore, miR-107 has a substantial role in the regulation of echinoderm microtubule-associated protein-like 4 (EML4) - anaplastic lymphoma kinase (ALK) fusion - which may result in the constitutive ALK activation, thus facilitating invasion, cell proliferation, and inhibition of apoptosis [42].

MiR-106a, as a member of the identified common miRNA families in this study, is an oncogenic miRNA which targets transcription factor (TF) of FOXO3 thus modulating the expression of genes implicated in apoptosis, cell cycle arrest, and autophagy. Furthermore, FOXO3a as a target gene of miR-103, can boost metastasis downstream of PI3K/AKT prohibition in collaboration with the WNT/β-catenin pathway in colon cancer. However, the final results of transcriptional activation or inactivation of FOXO are changeable depending on the context wherein that they occur, as it is a tumor suppressor within the context of PI3K/AKT signaling in neuroblastoma [43]. Another TF targeted by miR-106a is E2F2 belonging to the E2F family, which controls the cell cycle as well as the function of tumor suppressor proteins. On the other hand, the AKT3 protein is known as a critical regulator of the PI3K-AKT-mTOR pathway and miR-106a may decrease the activities of the PI3K/AKT pathway by suppressing the transcription function of E2F2 as well as down-regulation of AKT3.

D. Yang et al. [44] stated that the expression level of miR-103, which we extracted as a common effective miRNA between both diseases mentioned, decreases in NSCLC and COPD tissues, while it inversely correlates with tumor stage and size. Moreover, miR-103 can prohibit cell proliferation, reduce tumor volume and weight, and increase apoptosis in NSCLC, while targeting MAP2K2 which is a member of MAPK signaling cascade and also a RAS downstream signaling pathway regulating cell proliferation, differentiation, and survival by ERK1/2 activation [45]. On the other hand, MAP2K2 as the downstream of proto-oncogene BRAF is also targeted by miR-17 which is up-regulated in NSCLC and COPD. MAP2K2 has a key role in cell proliferation and cell cycle regulation, so several compounds have been developed to inhibit it in various diseases [46].

MiR-17 also targets TGFA that encodes transforming growth factor-α (TGF-α), a member of the epidermal growth factor family, what causes the activation of a signaling pathway for cell proliferation, differentiation, and development; this is while the down-regulation of TGF-α due to overexpression of miR-17 may inhibit tumorigenesis and disease progression [47]. Furthermore, in [48], L. Chen et al. defined E2F3 as a transcriptional activator that is a target gene of miR-17 capable of increasing the cellular proliferation via boosting the G1/S transition; besides, the down-regulation of E2F3 might be due to the molecular mechanism employed by up-regulation of miR-17 as a tumor suppressor.

While MiR-15, the other common core miRNA between COPD and NSCLC, may also function as a tumor-suppressor through the down-regulation of PIK3R3, a gene can serve as a second messenger in growth signaling pathways and can induce cell cycle arrest in the G1-G0 phase and act as a tumorigenesis miRNA in NSCLC [49–51]. However, T. Yang et al. [52] reported that miR-15b may suppress cell proliferation and induce apoptosis to inhibit cell survival, that is it can inhibit cell proliferation and invasion through down-regulation of ATK3.

S. Lim and P. Kaldis [53] investigated the regulation of the cell cycle pathway in the cell growth and stated that cyclin dependent kinases (CDKs), which are the target genes of miR-17, miR-107, and miR-103, are the key regulatory enzymes that regulate the progression of cells. In addition, cyclin-CDK inhibitor (CKI) family members have involved various functions including DNA damage repair, transcription, metabolism, epigenetic regulation, proteolytic degradation, stem cell self-renewal, spermatogenesis, and neuronal functions [54]. Moreover, EM. Gordon et al. [55] reported that the transcription factors E2Fs and their regulator Rb are the targets of CDKs, while the E2F proteins during the G1 phase of the cell cycle are activated by phosphorylation of Rb by CDK4/cyclin D (cycD) and Cdk2/cyclin E (cycE) complexes. As such, the transcriptional regulator E2F was found to be a crucial transcriptional regulator in cell cycle [56]. According to our enrichment analysis results, E2F is the target of miR-15 and miR-106a, and it seems the dysregulation of CDKs by some regulator miRNAs could affect cell proliferation or division. Moreover, P53 and its transcriptional targets has a critical function in both G1 and G2 checkpoints [53]. Furthermore, the transcription of various miRNAs is activated by p53 resulting in the repression of genes regulating DNA repair, apoptosis, and cell-cycle progression. Moreover, the regulation of p53 by miR-17, miR-103a, and miR-103b in the cell cycle pathway may be effective in apoptosis. Therefore, the effects of these miRNAs on cell cycle can provide new perspectives on the treatment.

## Future work

This study has investigated the interactions between core miRNAs and target genes within enriched pathways through miRNA-pathway enrichment analysis in an attempt to identify miRNA biomarkers that are common between COPD and NSCLC. In addition to miRNA-target genes in each pathway, there exist other miRNAs that might have interactions with the miRNA-target genes in the pathways; thus, the study of gene co-expression networks between our identified target genes and genes within the pathways might be a very informative site in this study and can be considered as a research field for future work.

## Conclusion

In conclusion, this study showed that the identified miRNAs including miR-106a, miR-17, miR-17, miR-15b, miR-107, and miR-103 were dysregulated in COPD and NSCLC. Moreover, the identification of molecular functions and investigating common pathways regulated by the discovered miRNAs and their target genes may help to look into and understand the possible mechanisms at work in such diseases. As such, it also may have the potential to be used for diagnostic as well as therapeutic goals regarding both diseases.

## Methods

### MiRNA expression profiles

In this project, we considered two miRNA expression profiles related to COPD and NSCLC. Both datasets were downloaded from the gene expression omnibus database (https://www.ncbi.nlm.nih.gov/geo/). The first dataset with the accession number GSE38974 contained 70 miRNAs that are differentially expressed between COPD subjects and smokers without COPD. The used platform is a miRCURY LNA microarray (GPL7723) for the miRNA dataset. Accordingly, the differential miRNA expression set between control and COPD samples were identified using a statistical test with the false positive discovery rate (pFDR) lower than 0.05 and with at least 1.5 fold changes between the groups. Finally, the dataset contained the miRNA expression profiles of 27 samples of which 19 samples were related to the COPD patients and 8 samples were related to normal subjects.

The second dataset with the accession number GSE36681 contained miRNA expression profiling of 56 pairs of fresh-frozen (FF) and 47 pairs of formalin-fixed, paraffin-embedded (FFPE) samples taken from never smokers’ lungs. The most differentially expressed miRNAs were evaluated by Cox analysis and Log-Rank test. Then, the functional significance of the most significant miRNAs was then measured via detecting the candidate targets experimentally. The Illumina human microRNA expression beadchip was used as the platform for this experiment with GPL8179, what included 206 samples of which again the same numbers of samples, as stated above, were related to NSCLC patients and normal tissues. As such, the GEOquery R package [57] was used for downloading the expression data.

### Normalization and pre-processing of miRNA expression profiles

All expression data were quantile-normalized and log2-transformed in R using EdgeR package [58]. Afterward, the samples were checked to exclude the ones containing the missing data or zero variances. Two expression matrices related to COPD and NSCLC cases were reconstructed.

### miRNA set enrichment analysis

In the next step, both datasets were enriched using the MiRSEA package in R [59]. This package was utilized to pathway enrichment analysis of differential expressed miRNAs (DEMs) using KEGG pathways. It is to be mentioned that MiRSEA determines the miRNAs regulating pathways and calculates miRNA-pathway weights based on the hypergeometric test (eq. (1)).

1 $$ {W}_{ij}=1-{p}_{ij} $$

In this equation, *Wij* denotes the weight of association between miRNA *i* and pathway *j*, and *p* is measured as eq. 2.

2 $$ {p}_{ij}=\sum \limits_{x=r}^n\frac{\left(\begin{array}{c}t\\ {}x\end{array}\right)\left(\begin{array}{c}m-t\\ {}n-x\end{array}\right)}{\left(\begin{array}{c}m\\ {}n\end{array}\right)} $$

Where *m* denotes the number of genes in the whole genome; *t* is the number of genes involved in pathway *j*; *n* is the number of targets of miRNA *i*; *r* denotes the number of overlaps between targets of miRNA *i* and genes in pathway *j*.

MiRSEA determines DEMs between the two phenotypes considering FDR < 1 and it thus carries out enrichment analysis by comparing DEMs with the miRNAs list in various pathways. Following this, it combines the differential expression levels of the miRNAs and the miRNA-pathway weight (*Wij*), and defines a *miRScore* for each enriched miRNA-pathway as eq. (3).

3 $$ miRScore=\left(1+{W}_i\right)\times {DE}_i $$

Where *W*_*i*_ is the weight of miRNA *i* with a given pathway and *DE*_*i*_ is the differential expression level of miRNA *i*.

Thus, a miRNA in a pathway with *miRScore* greater than zero indicates that the miRNA would probably regulate the pathway in the specific phenotype. MiRSEA ranks miRNAs in the profile and forms miRNA list according to the decreasing *miRScore*. We selected core miRNAs with the highest *miRScores* in each pathway for COPD and NSCLC, each separately, as these core miRNAs may have key functions in their pathways through regulating their target genes.

### Discovering dysregulated pathways and common miRNAs between COPD and NSCLC

To identify dysregulated KEGG pathways, we first sorted all enriched pathways based on miRNA Enrichment Score (*miRES*) that shows the extent of overrepresentation of pathways toward top or bottom of the ranked miRNA list. For COPD and NSCLC, we mapped both ranked list of dysregulated pathways and selected miRNAs which either were common or had higher *miRESs*. Then, we identified canonical pathways associated with a specific phenotype. We discovered the regulated pathways by miRNA set that were common between COPD and NSCLC and also had the differential expression of miRNAs among the two phenotypes. Finally, we selected the most significant pathways and related miRNAs (miRNA-pathways) based on *ES.*

For each pathway in both diseases, we evaluated the miRNA-pathways to determine which miRNAs regulated the pathway with more targets. We determined the correlated miRNAs within each pathway with a differential weighted score (dw-score) based on eq. (1) for each disease, separately. Among these miRNAs, we specified core miRNAs at and before the point where *miRSEA* is acquired (*miRSEA(p)* < 0) and then selected common miRNAs between COPD and NSCLC. After identifying common core miRNAs between the two diseases, we found those miRNAs that were common among all selected pathways, and created two lists of miRNAs (COPD and NSCLC cases) for each enriched pathway. We then combined both miRNA lists related to the diseases for each common enriched pathway in order to preserve only joint core miRNAs in each list. Finally, we calculated the mean of enrichment scores for each pathway and reconstructed a list of common miRNAs among all enriched pathways. In order to select the most significant common miRNAs among all enriched pathways, we selected the miRNAs based on the highest of average enrichment score found in all enriched pathways.

### Predicting miRNA targets and analyzing significant common pathways

The target genes of the selected miRNAs were identified by MiRSEA through four target genes prediction databases, i.e. miRWalk, TarBase, miRTarBase, and miR2Disease. To better understand the regulation mechanisms of these common miRNAs within the enriched pathways, we mapped these targets into three numbers of the most common pathways. In addition, to visualize miRNAs and their target genes in a pathway, we used WikiPathway [60] and Pathvisio [61] aiming to map and analyze the miRNAs within pathways.

## Supplementary Information

**Additional file 1: Table S1.** Common core miRNAs among all enriched pathways. In addition, all miRNAs are depicted with color scales from Green for more replicated miRNAs to Yellow for less replicated miRNAs. For example, hsa-miR-107 is common between five pathways: cell cycle, ERBB signaling, p53 signaling, VEGF signaling, and non-small cell lung cancer pathways, thus is highlighted with dark green, or hsa-miR-203 is shared between two pathways: cell cycle and non -small cell lung cancer pathways which is specified with light yellow. **Table S2.** Down-regulated enriched pathways in COPD. Also, the size of pathways based on the number of contributed features (SIZE), pathways’ enrichment scores before and after running enrichment peak (ES and NES), percentage of miRNA list before running enrichment peak (Mir%), and enrichment signal strength are represented in the columns. Moreover, the strength of NESs for all pathways is depicted by color-scaled column, which means that the red NES is more meaningful pathway in biology than the green one. **Table S3.** Up-regulated enriched pathways in NSCLC. Also, the size of pathways based on the number of contributed features (SIZE), pathways’ enrichment scores before and after running enrichment peak (ES and NES), percentage of miRNA list before running enrichment peak (Mir%), and enrichment signal strength are represented in the columns. Moreover, the strength of NESs for all pathways is depicted by color-scaled column, which means that the red NES is more meaningful pathway in biology than the green one.

## Data Availability

The datasets used and/or analyzed during the current study are available at GEO database: https://www.ncbi.nlm.nih.gov/geo/query/acc.cgi?acc=GSE38974 and https://www.ncbi.nlm.nih.gov/geo/query/acc.cgi?acc=GSE36681. Programming language: R. Other requirements: R environment. R. Packages: GEOquery, EdgR, and MiRSEA. Tested on R version 3.6.1.

## Abbreviations

AKT: Serine/threonine-protein kinase
ALK: Anaplastic lymphoma kinase
AMPK: Adenosine monophosphate-activated protein kinase
ACC: Acetyl-CoA carboxylase
BDNF: Brain-derived neurotrophic factor
CDK: Cyclin-dependent kinase
CycD: CDK4/cyclin D
CycE: Cdk2/cyclin E
CKI: Cyclin-CDK inhibitor
COPD: Chronic obstructive pulmonary disease
DEM: Differential expressed miRNA
EGFR: Epidermal growth factor receptor
EML4: Echinoderm microtubule-associated protein-like 4
ERK1/2: Extracellular signal-regulated protein kinase
ES: Enrichment score
FDR: False discovery rate
FOXO3: Fork head box O3
FPKM: Fragments per kilo base of transcript per million mapped reads
GEO: Gene expression omnibus
MAP2K2: Dual specificity mitogen-activated protein kinase kinase 2
MAPK: Mitogen-activated protein kinase
Mir%: Percentage of miRNA list before running enrichment peak
MiRES: miRNA Enrichment Score
MiRSEA: MiRNA set enrichment analysis
MiRNAs: MicroRNAs
NSCLC: Non-small cell lung cancer
PI3K: Phosphatidylinositol-3 kinase
PIK3R3: Phosphatidylinositol 3-kinase regulatory subunit gamma
PKB: Protein kinase B
PCC: Pearson correlation coefficient
SCLC: Small cell lung cancer
TF: Transcription factor
TGF: Transforming growth factor
TrkB: Tropomyosin-related receptor kinase B
VEGF: Vascular endothelial growth factor
