# Supplementary material for: Identification of common microRNA between COPD and non-small cell lung cancer through pathway enrichment analysis
Source: BMC Genom Data. 2021 Oct 12;22:41. doi: 10.1186/s12863-021-00986-z (PMC8507163; doi:10.1186/s12863-021-00986-z)
Supplement: Supplementary file 1 — Additional file 1: Table S1. Common core miRNAs among all enriched pathways. In addition, all miRNAs are depicted with color scales from Green for more replicated miRNAs to Yellow for less replicated miRNAs. For example, hsa-miR-107 is common between five pathways: cell cycle, ERBB signaling, p53 signaling, VEGF signaling, and non-small cell lung cancer pathways, thus is highlighted with dark green, or hsa-miR-203 is shared between two pathways: cell cycle and non -small cell lung cancer pathways which is specified with light yellow. Table S2. Down-regulated enriched pathways in COPD. Also, the size of pathways based on the number of contributed features (SIZE), pathways’ enrichment scores before and after running enrichment peak (ES and NES), percentage of miRNA list before running enrichment peak (Mir%), and enrichment signal strength are represented in the columns. Moreover, the strength of NESs for all pathways is depicted by color-scaled column, which means that the red NES is more meaningful pathway in biology than the green one. Table S3. Up-regulated enriched pathways in NSCLC. Also, the size of pathways based on the number of contributed features (SIZE), pathways’ enrichment scores before and after running enrichment peak (ES and NES), percentage of miRNA list before running enrichment peak (Mir%), and enrichment signal strength are represented in the columns. Moreover, the strength of NESs for all pathways is depicted by color-scaled column, which means that the red NES is more meaningful pathway in biology than the green one. [file 12863_2021_986_MOESM1_ESM.docx]

| KEGG_CELL_  CYCLE | KEGG_ERBB_  SIGNALING | KEGG_P53_  SIGNALING | KEGG_TGF_  BETA_SIGNALING | KEGG_VEGF_  SIGNALING | KEGG_WNT_  SIGNALING | KEGG_NON_SMALL_  CELL_LUNG_CANCER |
| --- | --- | --- | --- | --- | --- | --- |
| hsa-miR-107 | hsa-let-7b | hsa-miR-107 | hsa-miR-133a | hsa-miR-17 | hsa-miR-17 | hsa-miR-103 |
| hsa-miR-654-3p | hsa-let-7c | hsa-let-7d | hsa-let-7c | hsa-miR-107 | hsa-miR-15a | hsa-let-7c |
| hsa-let-7d | hsa-miR-1 | hsa-miR-654-3p | hsa-miR-1 | hsa-let-7b | hsa-let-7c | hsa-miR-17 |
| hsa-miR-1 | hsa-miR-654-3p | hsa-miR-1 | hsa-miR-455-3p | hsa-let-7d | hsa-miR-133a | hsa-miR-455-3p |
| hsa-miR-455-3p | hsa-miR-193a-3p | hsa-miR-361-5p | hsa-let-7d | hsa-miR-16 | hsa-miR-103 | hsa-miR-106a |
| hsa-miR-17 | hsa-miR-107 | hsa-let-7c |  | hsa-miR-193a-3p | hsa-let-7b | hsa-miR-107 |
| hsa-miR-15b | hsa-miR-17 | hsa-miR-15a |  | hsa-miR-133a | hsa-miR-1285 | hsa-miR-1285 |
| hsa-miR-103 | hsa-let-7d | hsa-miR-133a |  |  | hsa-miR-1 | hsa-miR-133a |
| hsa-miR-1285 | hsa-miR-133b | hsa-miR-15b |  |  | hsa-let-7e | hsa-miR-133b |
| hsa-let-7b | hsa-miR-106a | hsa-let-7b |  |  |  | hsa-miR-15b |
| hsa-let-7e |  | hsa-miR-103 |  |  |  | hsa-miR-203 |
| hsa-miR-106a |  | hsa-miR-106a |  |  |  |  |
| hsa-miR-361-5p |  | hsa-miR-17 |  |  |  |  |
| hsa-miR-203 |  | hsa-miR-16 |  |  |  |  |
| hsa-let-7c |  | hsa-miR-1285 |  |  |  |  |

Table S1. Common core miRNAs among all enriched pathways. In addition, all miRNAs are depicted with color scales from Green for more replicated miRNAs to Yellow for less replicated miRNAs. For example, hsa-miR-107 is common between five pathways: cell cycle, ERBB signaling, p53 signaling, VEGF signaling, and ‌ non-small cell lung cancer pathways, thus is highlighted with dark green, or hsa-miR-203 is shared between two pathways: cell cycle and non -small cell lung cancer pathways which is specified with light yellow.

Table S2. Down-regulated enriched pathways in COPD. Also, the size of pathways based on the number of contributed features (SIZE), pathways’ enrichment scores before and after running enrichment peak (ES and NES), percentage of miRNA list before running enrichment peak (Mir%), and enrichment signal strength are represented in the columns. Moreover, the strength of NESs for all pathways is depicted by color-scaled column, which means that the red NES is more meaningful pathway in biology than the green one.

| Pathway | SIZE | ES | NES | Mir \% | Signal |
| --- | --- | --- | --- | --- | --- |
| KEGG_OOCYTE_MEIOSIS | 54 | -0.76904 | -2.6434 | 0.0726 | 0.502 |
| KEGG_REGULATION_OF_ACTIN_CYTOSKELETON | 85 | -0.71525 | -2.5143 | 0.0826 | 0.45 |
| KEGG_CELL_CYCLE | 124 | -0.66193 | -2.4711 | 0.125 | 0.46 |
| KEGG_RENAL_CELL_CARCINOMA | 108 | -0.67663 | -2.4694 | 0.115 | 0.447 |
| KEGG_NON_SMALL_CELL_LUNG_CANCER | 105 | -0.64361 | -2.407 | 0.121 | 0.464 |
| KEGG_ERBB_SIGNALING_PATHWAY | 97 | -0.58372 | -2.3976 | 0.115 | 0.414 |
| KEGG_P53_SIGNALING_PATHWAY | 92 | -0.66285 | -2.396 | 0.115 | 0.455 |
| KEGG_VEGF_SIGNALING_PATHWAY | 68 | -0.59528 | -2.3886 | 0.108 | 0.415 |
| KEGG_TGF_BETA_SIGNALING_PATHWAY | 66 | -0.65517 | -2.3876 | 0.113 | 0.453 |
| KEGG_WNT_SIGNALING_PATHWAY | 32 | -0.73266 | -2.383 | 0.0657 | 0.539 |
| KEGG_MELANOGENESIS | 52 | -0.72139 | -2.3704 | 0.106 | 0.52 |
| KEGG_SMALL_CELL_LUNG_CANCER | 108 | -0.67663 | -2.3694 | 0.115 | 0.447 |
| KEGG_VASCULAR_SMOOTH_MUSCLE_CONTRACTION | 39 | -0.79367 | -2.3686 | 0.081 | 0.534 |
| KEGG_INSULIN_SIGNALING_PATHWAY | 77 | -0.68847 | -2.343 | 0.112 | 0.465 |
| KEGG_PATHWAYS_IN_CANCER | 182 | -0.63089 | -2.3398 | 0.133 | 0.432 |
| KEGG_PROGESTERONE_MEDIATED_OOCYTE_MATURATION | 71 | -0.6767 | -2.3226 | 0.115 | 0.461 |
| KEGG_CHEMOKINE_SIGNALING_PATHWAY | 81 | -0.68209 | -2.3093 | 0.109 | 0.446 |
| KEGG_BLADDER_CANCER | 105 | -0.64361 | -2.307 | 0.121 | 0.464 |
| KEGG_DILATED_CARDIOMYOPATHY | 36 | -0.79032 | -2.3034 | 0.0765 | 0.554 |
| KEGG_VIRAL_MYOCARDITIS | 59 | -0.59376 | -2.3022 | 0.139 | 0.596 |
| KEGG_PANCREATIC_CANCER | 112 | -0.64492 | -2.3019 | 0.119 | 0.439 |
| KEGG_APOPTOSIS | 70 | -0.65003 | -2.2985 | 0.108 | 0.417 |
| KEGG_FC_GAMMA_R_MEDIATED_PHAGOCYTOSIS | 56 | -0.68795 | -2.2865 | 0.0795 | 0.412 |
| KEGG_PYRIMIDINE_METABOLISM | 32 | -0.73266 | -2.283 | 0.0657 | 0.539 |
| KEGG_GLIOMA | 108 | -0.61591 | -2.2631 | 0.123 | 0.425 |
| KEGG_PATHOGENIC_ESCHERICHIA_COLI_INFECTION | 52 | -0.72868 | -2.2602 | 0.0734 | 0.427 |
| KEGG_GAP_JUNCTION | 58 | -0.68736 | -2.2602 | 0.106 | 0.468 |
| KEGG_PROSTATE_CANCER | 138 | -0.62867 | -2.2559 | 0.119 | 0.421 |
| KEGG_ALZHEIMERS_DISEASE | 65 | -0.62858 | -2.2558 | 0.117 | 0.415 |
| KEGG_NEUROTROPHIN_SIGNALING_PATHWAY | 105 | -0.63937 | -2.2545 | 0.112 | 0.441 |
| KEGG_CITRATE_CYCLE_TCA_CYCLE | 20 | -0.75189 | -2.2426 | 0.0635 | 0.571 |
| KEGG_AXON_GUIDANCE | 77 | -0.62605 | -2.2351 | 0.109 | 0.418 |
| KEGG_SPLICEOSOME | 41 | -0.67348 | -2.2338 | 0.068 | 0.446 |
| KEGG_LEISHMANIA_INFECTION | 39 | -0.7411 | -2.2303 | 0.0665 | 0.518 |
| KEGG_PURINE_METABOLISM | 37 | -0.73149 | -2.2216 | 0.0711 | 0.517 |
| KEGG_TOLL_LIKE_RECEPTOR_SIGNALING_PATHWAY | 55 | -0.66886 | -2.2213 | 0.0635 | 0.427 |
| KEGG_FOCAL_ADHESION | 130 | -0.62503 | -2.2163 | 0.131 | 0.423 |
| KEGG_HYPERTROPHIC_CARDIOMYOPATHY_HCM | 32 | -0.75442 | -2.2145 | 0.0742 | 0.504 |
| KEGG_ANTIGEN_PROCESSING_AND_PRESENTATION | 32 | -0.7264 | -2.2076 | 0.0933 | 0.552 |
| KEGG_MELANOMA | 114 | -0.60834 | -2.2027 | 0.123 | 0.421 |
| KEGG_TIGHT_JUNCTION | 79 | -0.63315 | -2.2009 | 0.112 | 0.407 |
| KEGG_ENDOCYTOSIS | 76 | -0.6155 | -2.199 | 0.125 | 0.416 |
| KEGG_HEDGEHOG_SIGNALING_PATHWAY | 26 | -0.77734 | -2.1986 | 0.0543 | 0.52 |
| KEGG_THYROID_CANCER | 62 | -0.64925 | -2.1958 | 0.109 | 0.438 |
| KEGG_COLORECTAL_CANCER | 100 | -0.60399 | -2.1948 | 0.119 | 0.401 |
| KEGG_CHRONIC_MYELOID_LEUKEMIA | 117 | -0.60803 | -2.1882 | 0.119 | 0.405 |
| KEGG_CARDIAC_MUSCLE_CONTRACTION | 30 | -0.74243 | -2.1748 | 0.0673 | 0.477 |
| KEGG_HUNTINGTONS_DISEASE | 63 | -0.64457 | -2.1721 | 0.0872 | 0.396 |
| KEGG_GNRH_SIGNALING_PATHWAY | 57 | -0.67446 | -2.1713 | 0.106 | 0.443 |
| KEGG_ADHERENS_JUNCTION | 81 | -0.61856 | -2.1711 | 0.118 | 0.418 |
| KEGG_OXIDATIVE_PHOSPHORYLATION | 34 | -0.68941 | -2.1678 | 0.0772 | 0.418 |
| KEGG_CYSTEINE_AND_METHIONINE_METABOLISM | 27 | -0.73017 | -2.1314 | 0.0344 | 0.438 |
| KEGG_ENDOMETRIAL_CANCER | 95 | -0.58421 | -2.1282 | 0.112 | 0.393 |
| KEGG_N_GLYCAN_BIOSYNTHESIS | 23 | -0.72721 | -2.1184 | 0.0627 | 0.539 |
| KEGG_MAPK_SIGNALING_PATHWAY | 109 | -0.57736 | -2.1164 | 0.129 | 0.401 |
| KEGG_UBIQUITIN_MEDIATED_PROTEOLYSIS | 52 | -0.64617 | -2.115 | 0.138 | 0.449 |
| KEGG_LONG_TERM_DEPRESSION | 41 | -0.6558 | -2.1032 | 0.113 | 0.469 |
| KEGG_ALLOGRAFT_REJECTION | 12 | -0.85566 | -2.1021 | 0.0482 | 0.64 |
| KEGG_NUCLEOTIDE_EXCISION_REPAIR | 17 | -0.80731 | -2.096 | 0.0612 | 0.56 |
| KEGG_GLUTATHIONE_METABOLISM | 14 | -0.79507 | -2.0921 | 0.0589 | 0.544 |
| KEGG_CALCIUM_SIGNALING_PATHWAY | 48 | -0.64013 | -2.0914 | 0.112 | 0.442 |
| KEGG_NOD_LIKE_RECEPTOR_SIGNALING_PATHWAY | 40 | -0.6541 | -2.0911 | 0.0657 | 0.41 |
| KEGG_ARRHYTHMOGENIC_RIGHT_VENTRICULAR_CARDIOMYOPATHY | 30 | -0.68879 | -2.0851 | 0.0635 | 0.447 |
| KEGG_GLYCEROPHOSPHOLIPID_METABOLISM | 17 | -0.78244 | -2.0762 | 0.0673 | 0.5 |
| KEGG_LEUKOCYTE_TRANSENDOTHELIAL_MIGRATION | 58 | -0.63415 | -2.0651 | 0.0856 | 0.396 |
| KEGG_LYSINE_DEGRADATION | 24 | -0.72194 | -2.0576 | 0.0627 | 0.477 |
| KEGG_ACUTE_MYELOID_LEUKEMIA | 78 | -0.58943 | -2.0542 | 0.107 | 0.365 |
| KEGG_LONG_TERM_POTENTIATION | 43 | -0.63961 | -2.0517 | 0.112 | 0.427 |
| KEGG_PARKINSONS_DISEASE | 37 | -0.64896 | -2.0472 | 0.114 | 0.444 |
| KEGG_JAK_STAT_SIGNALING_PATHWAY | 73 | -0.58805 | -2.0401 | 0.156 | 0.404 |
| KEGG_AUTOIMMUNE_THYROID_DISEASE | 12 | -0.83686 | -2.0312 | 0.0581 | 0.634 |
| KEGG_SYSTEMIC_LUPUS_ERYTHEMATOSUS | 32 | -0.68765 | -2.0261 | 0.0642 | 0.45 |
| KEGG_GRAFT_VERSUS_HOST_DISEASE | 12 | -0.80168 | -2.0126 | 0.0512 | 0.559 |
| KEGG_DORSO_VENTRAL_AXIS_FORMATION | 36 | -0.62778 | -2.0029 | 0.105 | 0.46 |
| KEGG_TYPE_II_DIABETES_MELLITUS | 40 | -0.62894 | -1.9928 | 0.0528 | 0.342 |
| KEGG_MTOR_SIGNALING_PATHWAY | 49 | -0.6238 | -1.9804 | 0.0994 | 0.401 |
| KEGG_TYPE_I_DIABETES_MELLITUS | 14 | -0.81947 | -1.9748 | 0.055 | 0.614 |
| KEGG_CELL_ADHESION_MOLECULES_CAMS | 33 | -0.66341 | -1.9732 | 0.0742 | 0.461 |
| KEGG_PRION_DISEASES | 30 | -0.68024 | -1.9721 | 0.0772 | 0.472 |
| KEGG_FC_EPSILON_RI_SIGNALING_PATHWAY | 48 | -0.58702 | -1.9693 | 0.112 | 0.365 |
| KEGG_VASOPRESSIN_REGULATED_WATER_REABSORPTION | 21 | -0.72377 | -1.968 | 0.0512 | 0.551 |
| KEGG_RNA_DEGRADATION | 20 | -0.69906 | -1.9639 | 0.1 | 0.457 |
| KEGG_HEMATOPOIETIC_CELL_LINEAGE | 25 | -0.68007 | -1.9615 | 0.118 | 0.432 |
| KEGG_B_CELL_RECEPTOR_SIGNALING_PATHWAY | 57 | -0.58854 | -1.9597 | 0.111 | 0.375 |
| KEGG_BASAL_CELL_CARCINOMA | 35 | -0.64385 | -1.9566 | 0.223 | 0.456 |
| KEGG_BASAL_TRANSCRIPTION_FACTORS | 19 | -0.71254 | -1.9482 | 0.0772 | 0.542 |
| KEGG_RIG_I_LIKE_RECEPTOR_SIGNALING_PATHWAY | 25 | -0.66846 | -1.9476 | 0.039 | 0.431 |
| KEGG_CYTOKINE_CYTOKINE_RECEPTOR_INTERACTION | 71 | -0.57527 | -1.9382 | 0.109 | 0.385 |
| KEGG_PYRUVATE_METABOLISM | 19 | -0.71752 | -1.9341 | 0.0787 | 0.492 |
| KEGG_NEUROACTIVE_LIGAND_RECEPTOR_INTERACTION | 21 | -0.66233 | -1.9314 | 0.0612 | 0.409 |
| KEGG_EPITHELIAL_CELL_SIGNALING_IN_HELICOBACTER_PYLORI_INFECTION | 53 | -0.57309 | -1.9302 | 0.138 | 0.407 |
| KEGG_PEROXISOME | 17 | -0.7384 | -1.9272 | 0.068 | 0.444 |
| KEGG_ALANINE_ASPARTATE_AND_GLUTAMATE_METABOLISM | 17 | -0.72272 | -1.9207 | 0.166 | 0.597 |
| KEGG_DNA_REPLICATION | 15 | -0.72498 | -1.9103 | 0.0604 | 0.57 |
| KEGG_ECM_RECEPTOR_INTERACTION | 37 | -0.62648 | -1.9077 | 0.109 | 0.396 |
| KEGG_VIBRIO_CHOLERAE_INFECTION | 28 | -0.6926 | -1.9074 | 0.124 | 0.448 |
| KEGG_CYTOSOLIC_DNA_SENSING_PATHWAY | 20 | -0.68733 | -1.9071 | 0.0872 | 0.464 |
| KEGG_INTESTINAL_IMMUNE_NETWORK_FOR_IGA_PRODUCTION | 13 | -0.77859 | -1.8988 | 0.0925 | 0.635 |
| KEGG_ARACHIDONIC_ACID_METABOLISM | 12 | -0.76268 | -1.8896 | 0.0986 | 0.531 |
| KEGG_MISMATCH_REPAIR | 11 | -0.77744 | -1.8707 | 0.0596 | 0.603 |
| KEGG_T_CELL_RECEPTOR_SIGNALING_PATHWAY | 72 | -0.53757 | -1.8586 | 0.114 | 0.378 |
| KEGG_PRIMARY_IMMUNODEFICIENCY | 10 | -0.83108 | -1.8541 | 0.0428 | 0.579 |
| KEGG_AMYOTROPHIC_LATERAL_SCLEROSIS_ALS | 46 | -0.59139 | -1.8533 | 0.0849 | 0.412 |
| KEGG_LYSOSOME | 27 | -0.6549 | -1.8532 | 0.0772 | 0.419 |
| KEGG_NATURAL_KILLER_CELL_MEDIATED_CYTOTOXICITY | 57 | -0.55759 | -1.8493 | 0.0635 | 0.275 |
| KEGG_GLYCOLYSIS_GLUCONEOGENESIS | 36 | -0.63318 | -1.8472 | 0.187 | 0.487 |
| KEGG_COMPLEMENT_AND_COAGULATION_CASCADES | 15 | -0.7304 | -1.8374 | 0.0291 | 0.327 |
| KEGG_AMINOACYL_TRNA_BIOSYNTHESIS | 17 | -0.67447 | -1.8346 | 0.151 | 0.506 |
| KEGG_PROPANOATE_METABOLISM | 15 | -0.68569 | -1.8277 | 0.0703 | 0.439 |
| KEGG_ARGININE_AND_PROLINE_METABOLISM | 21 | -0.6271 | -1.816 | 0.218 | 0.454 |
| KEGG_ABC_TRANSPORTERS | 20 | -0.6608 | -1.8039 | 0.0956 | 0.459 |
| KEGG_BIOSYNTHESIS_OF_UNSATURATED_FATTY_ACIDS | 13 | -0.73412 | -1.8027 | 0.115 | 0.619 |
| KEGG_AMINO_SUGAR_AND_NUCLEOTIDE_SUGAR_METABOLISM | 23 | -0.64151 | -1.8012 | 0.0734 | 0.41 |
| KEGG_RNA_POLYMERASE | 15 | -0.71753 | -1.7996 | 0.0772 | 0.56 |
| KEGG_ALDOSTERONE_REGULATED_SODIUM_REABSORPTION | 39 | -0.57253 | -1.7996 | 0.118 | 0.35 |
| KEGG_TASTE_TRANSDUCTION | 10 | -0.77076 | -1.7911 | 0.0474 | 0.48 |
| KEGG_PROTEASOME | 22 | -0.66735 | -1.7889 | 0.0321 | 0.358 |
| KEGG_ADIPOCYTOKINE_SIGNALING_PATHWAY | 39 | -0.57723 | -1.7842 | 0.042 | 0.304 |
| KEGG_INOSITOL_PHOSPHATE_METABOLISM | 35 | -0.58902 | -1.7816 | 0.119 | 0.414 |
| KEGG_VALINE_LEUCINE_AND_ISOLEUCINE_BIOSYNTHESIS | 12 | -0.72538 | -1.7764 | 0.0306 | 0.489 |
| KEGG_PHOSPHATIDYLINOSITOL_SIGNALING_SYSTEM | 38 | -0.55978 | -1.7702 | 0.0742 | 0.326 |
| KEGG_GLYCINE_SERINE_AND_THREONINE_METABOLISM | 10 | -0.76926 | -1.7395 | 0.0673 | 0.564 |
| KEGG_PENTOSE_PHOSPHATE_PATHWAY | 24 | -0.60949 | -1.734 | 0.0596 | 0.319 |
| KEGG_METABOLISM_OF_XENOBIOTICS_BY_CYTOCHROME_P450 | 10 | -0.7485 | -1.7208 | 0.0466 | 0.48 |
| KEGG_HOMOLOGOUS_RECOMBINATION | 13 | -0.72329 | -1.711 | 0.0757 | 0.503 |
| KEGG_FRUCTOSE_AND_MANNOSE_METABOLISM | 24 | -0.60747 | -1.7052 | 0.0482 | 0.364 |
| KEGG_NOTCH_SIGNALING_PATHWAY | 35 | -0.53065 | -1.6777 | 0.065 | 0.329 |
| KEGG_SELENOAMINO_ACID_METABOLISM | 13 | -0.63361 | -1.6472 | 0.0306 | 0.377 |
| KEGG_DRUG_METABOLISM_OTHER_ENZYMES | 11 | -0.7036 | -1.6425 | 0.0321 | 0.444 |
| KEGG_PORPHYRIN_AND_CHLOROPHYLL_METABOLISM | 14 | -0.6961 | -1.6276 | 0.117 | 0.51 |
| KEGG_STARCH_AND_SUCROSE_METABOLISM | 17 | -0.63572 | -1.623 | 0.0359 | 0.402 |
| KEGG_PROTEIN_EXPORT | 16 | -0.63383 | -1.5963 | 0.136 | 0.492 |
| KEGG_GLYCEROLIPID_METABOLISM | 13 | -0.66374 | -1.5844 | 0.0581 | 0.366 |
| KEGG_RIBOSOME | 40 | -0.50956 | -1.5502 | 0.114 | 0.366 |
| KEGG_NITROGEN_METABOLISM | 10 | -0.66689 | -1.5345 | 0.135 | 0.523 |
| KEGG_BUTANOATE_METABOLISM | 12 | -0.63017 | -1.5334 | 0.0619 | 0.473 |
| KEGG_SPHINGOLIPID_METABOLISM | 10 | -0.65884 | -1.5072 | 0.213 | 0.635 |
| KEGG_PPAR_SIGNALING_PATHWAY | 27 | -0.50863 | -1.4713 | 0.0757 | 0.385 |
| KEGG_GALACTOSE_METABOLISM | 22 | -0.50458 | -1.4469 | 0.0734 | 0.3 |
| KEGG_BASE_EXCISION_REPAIR | 19 | -0.54463 | -1.4455 | 0.0703 | 0.298 |
| KEGG_NON_HOMOLOGOUS_END_JOINING | 12 | -0.57255 | -1.3856 | 0.0872 | 0.461 |
| KEGG_TRYPTOPHAN_METABOLISM | 11 | -0.60391 | -1.351 | 0.181 | 0.525 |
| KEGG_SNARE_INTERACTIONS_IN_VESICULAR_TRANSPORT | 11 | -0.52751 | -1.3119 | 0.179 | 0.452 |
| KEGG_BETA_ALANINE_METABOLISM | 10 | -0.51139 | -1.2088 | 0.205 | 0.481 |
| KEGG_VALINE_LEUCINE_AND_ISOLEUCINE_DEGRADATION | 11 | -0.50359 | -1.1882 | 0.241 | 0.487 |
| KEGG_OLFACTORY_TRANSDUCTION | 11 | -0.48517 | -1.1789 | 0.196 | 0.368 |
| KEGG_ONE_CARBON_POOL_BY_FOLATE | 14 | -0.46836 | -1.1573 | 0.094 | 0.392 |

Table S3. Up-regulated enriched pathways in NSCLC. Also, the size of pathways based on the number of contributed features (SIZE), pathways’ enrichment scores before and after running enrichment peak (ES and NES), percentage of miRNA list before running enrichment peak (Mir%), and enrichment signal strength are represented in the columns. Moreover, the strength of NESs for all pathways is depicted by color-scaled column, which means that the red NES is more meaningful pathway in biology than the green one.

| Pathway | SIZE | ES | NES | Mir \% | Signal |
| --- | --- | --- | --- | --- | --- |
| KEGG_PRIMARY_IMMUNODEFICIENCY | 10 | 0.83905 | 1.8172 | 0.00556 | 0.303 |
| KEGG_P53_SIGNALING_PATHWAY | 100 | 0.49347 | 1.6518 | 0.127 | 0.325 |
| KEGG_ERBB_SIGNALING_PATHWAY | 90 | 0.47255 | 1.6308 | 0.102 | 0.285 |
| KEGG_NON_SMALL_CELL_LUNG_CANCER | 86 | 0.48247 | 1.6235 | 0.089 | 0.253 |
| KEGG_CELL_CYCLE | 116 | 0.4208 | 1.5168 | 0.106 | 0.267 |
| KEGG_APOPTOSIS | 67 | 0.46177 | 1.4878 | 0.132 | 0.343 |
| KEGG_WNT_SIGNALING_PATHWAY | 87 | 0.40255 | 1.382 | 0.124 | 0.321 |
| KEGG_PRION_DISEASES | 27 | 0.51335 | 1.3818 | 0.113 | 0.273 |
| KEGG_VEGF_SIGNALING_PATHWAY | 62 | 0.37869 | 1.3925 | 0.128 | 0.277 |
| KEGG_TGF_BETA_SIGNALING_PATHWAY | 60 | 0.37867 | 1.2741 | 0.0209 | 0.16 |
| KEGG_GLIOMA | 101 | 0.45883 | 1.1571 | 0.113 | 0.276 |
| KEGG_FOCAL_ADHESION | 122 | 0.43345 | 1.1566 | 0.127 | 0.328 |
| KEGG_THYROID_CANCER | 56 | 0.51962 | 1.1564 | 0.118 | 0.342 |
| KEGG_AXON_GUIDANCE | 72 | 0.48468 | 1.1558 | 0.146 | 0.382 |
| KEGG_VIRAL_MYOCARDITIS | 56 | 0.49721 | 1.1544 | 0.0709 | 0.27 |
| KEGG_COLORECTAL_CANCER | 94 | 0.45535 | 1.1496 | 0.111 | 0.305 |
| KEGG_CELL_ADHESION_MOLECULES_CAMS | 32 | 0.40792 | 1.1492 | 0.134 | 0.312 |
| KEGG_METABOLISM_OF_XENOBIOTICS_BY_CYTOCHROME_P450 | 10 | 0.66139 | 1.1458 | 0.0167 | 0.299 |
| KEGG_MAPK_SIGNALING_PATHWAY | 104 | 0.41645 | 1.1457 | 0.117 | 0.298 |
| KEGG_TRYPTOPHAN_METABOLISM | 10 | 0.69623 | 1.1439 | 0.0626 | 0.285 |
| KEGG_SMALL_CELL_LUNG_CANCER | 102 | 0.41316 | 1.1427 | 0.124 | 0.3 |
| KEGG_ENDOMETRIAL_CANCER | 88 | 0.40665 | 1.1405 | 0.115 | 0.286 |
| KEGG_CHRONIC_MYELOID_LEUKEMIA | 110 | 0.40288 | 1.1401 | 0.103 | 0.25 |
| KEGG_MELANOMA | 107 | 0.40221 | 1.1399 | 0.117 | 0.272 |
| KEGG_BLADDER_CANCER | 97 | 0.42573 | 1.1386 | 0.104 | 0.267 |
| KEGG_JAK_STAT_SIGNALING_PATHWAY | 69 | 0.42394 | 1.1379 | 0.0974 | 0.26 |
| KEGG_CYTOKINE_CYTOKINE_RECEPTOR_INTERACTION | 68 | 0.44052 | 1.1378 | 0.113 | 0.288 |
| KEGG_GLYCINE_SERINE_AND_THREONINE_METABOLISM | 10 | 0.5111 | 1.1377 | 0.0389 | 0.292 |
| KEGG_AUTOIMMUNE_THYROID_DISEASE | 12 | 0.59511 | 1.1374 | 0.064 | 0.238 |
| KEGG_PROTEIN_EXPORT | 14 | 0.59324 | 1.1361 | 0.057 | 0.275 |
| KEGG_NEUROTROPHIN_SIGNALING_PATHWAY | 100 | 0.40773 | 1.136 | 0.1 | 0.272 |
| KEGG_PANCREATIC_CANCER | 105 | 0.38932 | 1.1354 | 0.0946 | 0.252 |
| KEGG_LONG_TERM_DEPRESSION | 37 | 0.46173 | 1.1349 | 0.0682 | 0.239 |
| KEGG_ENDOCYTOSIS | 73 | 0.33642 | 1.1343 | 0.122 | 0.268 |
| KEGG_INTESTINAL_IMMUNE_NETWORK_FOR_IGA_PRODUCTION | 13 | 0.49293 | 1.1343 | 0.0125 | 0.155 |
| KEGG_BIOSYNTHESIS_OF_UNSATURATED_FATTY_ACIDS | 13 | 0.59247 | 1.134 | 0.0626 | 0.367 |
| KEGG_REGULATION_OF_ACTIN_CYTOSKELETON | 80 | 0.33585 | 1.1328 | 0.118 | 0.26 |
| KEGG_GLUTATHIONE_METABOLISM | 13 | 0.58162 | 1.1317 | 0.0584 | 0.369 |
| KEGG_HEMATOPOIETIC_CELL_LINEAGE | 22 | 0.51571 | 1.1306 | 0.00834 | 0.139 |
| KEGG_SPLICEOSOME | 39 | 0.44502 | 1.128 | 0.131 | 0.33 |
| KEGG_GALACTOSE_METABOLISM | 20 | 0.4823 | 1.1279 | 0.11 | 0.32 |
| KEGG_RENAL_CELL_CARCINOMA | 76 | 0.38328 | 1.1272 | 0.118 | 0.285 |
| KEGG_NATURAL_KILLER_CELL_MEDIATED_CYTOTOXICITY | 52 | 0.39569 | 1.1264 | 0.106 | 0.278 |
| KEGG_PYRIMIDINE_METABOLISM | 31 | 0.38885 | 1.1264 | 0.125 | 0.324 |
| KEGG_BUTANOATE_METABOLISM | 12 | 0.56703 | 1.1252 | 0.0723 | 0.314 |
| KEGG_AMINO_SUGAR_AND_NUCLEOTIDE_SUGAR_METABOLISM | 21 | 0.49553 | 1.1251 | 0.111 | 0.392 |
| KEGG_ARGININE_AND_PROLINE_METABOLISM | 19 | 0.53622 | 1.1246 | 0.0542 | 0.307 |
| KEGG_NOTCH_SIGNALING_PATHWAY | 34 | 0.43611 | 1.1235 | 0.0737 | 0.229 |
| KEGG_ARACHIDONIC_ACID_METABOLISM | 12 | 0.56603 | 1.1226 | 0.0167 | 0.333 |
| KEGG_OOCYTE_MEIOSIS | 51 | 0.38412 | 1.1226 | 0.138 | 0.4 |
| KEGG_FC_EPSILON_RI_SIGNALING_PATHWAY | 44 | 0.41488 | 1.1226 | 0.0751 | 0.224 |
| KEGG_PROGESTERONE_MEDIATED_OOCYTE_MATURATION | 67 | 0.38536 | 1.1202 | 0.142 | 0.339 |
| KEGG_RIBOSOME | 37 | 0.38927 | 1.1172 | 0.0765 | 0.263 |
| KEGG_N_GLYCAN_BIOSYNTHESIS | 21 | 0.45771 | 1.117 | 0.057 | 0.231 |
| KEGG_BASAL_TRANSCRIPTION_FACTORS | 19 | 0.4487 | 1.1125 | 0.113 | 0.288 |
| KEGG_LYSOSOME | 25 | 0.40916 | 1.1123 | 0.0834 | 0.304 |
| KEGG_DRUG_METABOLISM_OTHER_ENZYMES | 11 | 0.48134 | 1.0811 | 0.0654 | 0.259 |
| KEGG_DORSO_VENTRAL_AXIS_FORMATION | 32 | 0.65148 | 1.0802 | 0.0612 | 0.307 |
| KEGG_ECM_RECEPTOR_INTERACTION | 33 | 0.37891 | 1.075 | 0.0821 | 0.204 |
| KEGG_AMYOTROPHIC_LATERAL_SCLEROSIS_ALS | 45 | 0.54217 | 1.0652 | 0.121 | 0.375 |
| KEGG_GLYCOLYSIS_GLUCONEOGENESIS | 32 | 0.38052 | 1.039 | 0.124 | 0.315 |
| KEGG_VIBRIO_CHOLERAE_INFECTION | 25 | 0.37466 | 1.0364 | 0.0626 | 0.233 |
| KEGG_FRUCTOSE_AND_MANNOSE_METABOLISM | 21 | 0.40222 | 1.0234 | 0.111 | 0.305 |
| KEGG_ALDOSTERONE_REGULATED_SODIUM_REABSORPTION | 35 | 0.37191 | 1.0223 | 0.0862 | 0.247 |
| KEGG_HYPERTROPHIC_CARDIOMYOPATHY_HCM | 31 | 0.39286 | 1.003 | 0.0751 | 0.218 |
| KEGG_BASE_EXCISION_REPAIR | 18 | 0.39076 | 0.98513 | 0.00974 | 0.113 |
| KEGG_RNA_DEGRADATION | 18 | 0.39098 | 0.94207 | 0.0584 | 0.215 |
| KEGG_STARCH_AND_SUCROSE_METABOLISM | 17 | 0.37014 | 0.93473 | 0.104 | 0.27 |
| KEGG_AMINOACYL_TRNA_BIOSYNTHESIS | 16 | 0.38847 | 0.89441 | 0.117 | 0.339 |
| KEGG_GLYCEROLIPID_METABOLISM | 13 | 0.40066 | 0.88021 | 0.0584 | 0.221 |
| KEGG_NUCLEOTIDE_EXCISION_REPAIR | 16 | 0.37286 | 0.87056 | 0.11 | 0.284 |
